# Effectiveness of Psychotherapy vs Antidepressants for Depression in Primary Care in India: A Randomized Pilot Trial

**DOI:** 10.1192/j.eurpsy.2025.795

**Published:** 2025-08-26

**Authors:** J. R Pozuelo, A. Lahiri, R. Singh, S. D. Hollon, N. Dongare, A. Khan, A. Shukla, M. Ghosh, A. Kushwah, A. Bhan, Y. Parashar, V. Patel

**Affiliations:** 1 Global Health and Social Medicine, Harvard University, Boston, United States; 2 Sangath, Bhopal, India; 3 Vanderbilt University, Nashville; 4 Harvard University, Boston, United States

## Abstract

**Introduction:**

Depression is commonly treated with psychotherapy or antidepressants, but predicting which intervention will work best for a given patient remains a challenge. This pilot trial compared the feasibility, acceptability, and effectiveness of psychotherapy based on behavioral activation (Healthy Activity Program, HAP) and antidepressant medication (fluoxetine) in a primary care setting in India.

**Objectives:**

The trial had three key objectives: (1) assess the feasibility and acceptability of a randomized pilot trial comparing HAP and fluoxetine; (2) collect outcome data to refine study instruments and the baseline assessment; and (3) evaluate the preliminary comparative effectiveness of psychotherapy versus medication.

**Methods:**

The pilot trial was conducted at eight primary health care centres in Madhya Pradesh (India) from August 2023 to February 2024. Eligible participants (aged 18+, PHQ-9 score ≥10) with moderate to severe depression were randomized to receive either HAP or fluoxetine. Feasibility was assessed by recruitment, retention, and adherence to study procedures. Acceptability was measured by adherence to interventions. Preliminary efficacy, as a secondary outcome, was assessed through changes in depressive symptoms (PHQ-9) from baseline to the 3-month follow-up.

**Results:**

76 participants were randomized, with primary endpoint data available for 63 (83%). Retention rates and study assessment completion were acceptable across both arms. Intervention adherence was high, with 79% (30/38 in HAP and ADM groups) completing the treatment per protocol (≥6 HAP sessions or 70% medication adherence). PHQ-9 scores improved significantly, with an average reduction from 15.02 at baseline to 6.73 at the 3-month follow-up, with no statistically significant differences between treatments. Full remission (PHQ-9 < 5) was achieved by 45.16% (28/62) of participants. Additionally, the pilot study identified logistical challenges and facilitators that will help refine the protocol for the larger trial.

**Conclusions:**

This pilot trial successfully demonstrated the feasibility and acceptability of the study design, procedures, and interventions. The preliminary data suggest that both HAP and Fluoxetine are viable treatments for moderate to severe depression in primary care settings in India. The findings will be instrumental in informing the design and implementation of a larger precision trial.

**Disclosure of Interest:**

None Declared

